# Osteogenic and Adipogenic Differentiation Potential of Oral Cancer Stem Cells May Offer New Treatment Modalities

**DOI:** 10.3390/ijms24054704

**Published:** 2023-02-28

**Authors:** Milica Jaksic Karisik, Milos Lazarevic, Dijana Mitic, Nadja Nikolic, Maja Milosevic Markovic, Drago Jelovac, Jelena Milasin

**Affiliations:** 1 Department of Human Genetics, School of Dental Medicine, University of Belgrade, Dr. Subotica 8, 11000 Belgrade, Serbia; 2 Clinic for Maxillofacial Surgery, School of Dental Medicine, University of Belgrade, Dr. Subotica 8, 11000 Belgrade, Serbia

**Keywords:** oral cancer, cancer stem cells, CD44, osteogenic differentiation, adipogenic differentiation, miRNA-21, miRNA-133 and miRNA-491

## Abstract

(1) Treatment failure of oral squamous cell carcinoma (OSCC) is generally due to the development of therapeutic resistance caused by the existence of cancer stem cells (CSCs), a small cell subpopulation with marked self-renewal and differentiation capacity. Micro RNAs, notably miRNA-21, appear to play an important role in OSCC carcinogenesis. Our objectives were to explore the multipotency of oral CSCs by estimating their differentiation capacity and assessing the effects of differentiation on stemness, apoptosis, and several miRNAs’ expression. (2) A commercially available OSCC cell line (SCC25) and five primary OSCC cultures generated from tumor tissues obtained from five OSCC patients were used in the experiments. Cells harboring CD44, a CSC marker, were magnetically separated from the heterogeneous tumor cell populations. The CD44^+^ cells were then subjected to osteogenic and adipogenic induction, and the specific staining was used for differentiation confirmation. The kinetics of the differentiation process was evaluated by qPCR analysis of osteogenic (Bone Morphogenetic Protein—*BMP4*, Runt-related Transcription Factor 2—*RUNX2*, Alkaline Phosphatase—*ALP*) and adipogenic (Fibroblast Activation Protein Alpha—*FAP*, *LIPIN*, Peroxisome Proliferator-activated Receptor Gamma—*PPARG*) markers on days 0, 7, 14, and 21. Embryonic markers (Octamer-binding Transcription Factor 4—*OCT4*, Sex Determining Region Y Box 2—*SOX2*, and *NANOG*) and micro RNAs (miRNA-21, miRNA-133, and miRNA-491) were also correspondingly evaluated by qPCR. An Annexin V assay was used to assess the potential cytotoxic effects of the differentiation process. (3) Following differentiation, the levels of markers for the osteo/adipo lineages showed a gradual increase from day 0 to day 21 in the CD44^+^ cultures, while stemness markers and cell viability decreased. The oncogenic miRNA-21 also followed the same pattern of gradual decrease along the differentiation process, while tumor suppressor miRNA-133 and miRNA-491 levels increased. (4) Following induction, the CSCs acquired the characteristics of the differentiated cells. This was accompanied by loss of stemness properties, a decrease of the oncogenic and concomitant, and an increase of tumor suppressor micro RNAs.

## 1. Introduction

Oral squamous cell carcinoma (OSCC) is a frequent and aggressive malignancy in the group of head and neck tumors, with a prevalence of over 630,000 new cases per year worldwide [1]. Despite advances in multimodal therapeutic strategies, the main problem remains the resistance to chemotherapy and biologic agents, resulting in a poor 5-year survival rate of only 50% [2,3]. Recent evidence suggests that one of the reasons for OSCC therapy failure is the presence of a small pluripotent cell subpopulation identified as “cancer stem cells” (CSCs), which are considered to have a tumor-initiating and self-renewal ability [4]. Studies also indicate that CSCs possess a remarkable differentiation capacity [5,6], and this characteristic could potentially be exploited for novel OSCC therapeutic modalities, as conventional therapy regimens may effectively treat the bulk tumor mass yet leave CSCs behind as a source for tumor recurrence upon treatment [7].

The transmembrane glycoprotein CD44 has been recognized as a characteristic CSC surface marker that may be used independently or in combination with other markers for the identification of CSCs in various cancers [6]. CSCs show a high level of expression of embryonic (Octamer-binding Transcription Factor 4—*OCT4*, Sex Determining Region Y Box 2—*SOX2*, and *NANOG*) stem cell markers and different micro RNAs (miRNAs) as well [7,8]. More specifically, the overexpression of CD44 has been shown to increase the expression of embryonic transcription factors OCT4, SOX2, and NANOG [9].

Several miRNAs have been involved in cancer development, acting either as tumor suppressors or as oncogenes [10]. MiRNAs are short (20 to 24 nucleotide) non-coding RNA molecules that regulate the expression of up to 50% of all protein-coding genes at the post-translational level [11]. They are involved in a variety of biological processes, such as cell proliferation, apoptosis, immunological response, etc. [12,13]. As oncogenes or tumor suppressors, miRNAs play a critical role in cancerogenesis via the regulation of self-renewal and apoptosis across CSC signaling pathways [14].

MiRNA-21 is an oncogenic miRNA that is overexpressed in a variety of malignancies, and in OSCCs, its overexpression has been correlated with poor prognosis [15]. The *NANOG* signaling axis has been shown to stimulate the overexpression of miRNA-21 and regulate cell growth and self-renewal in CD44^+^ cells [16,17].

However, the present literature did not investigate the difference in the miRNA-21 expression between CSCs and the heterogenic cancer cell population or whether CSC differentiation affects the expression of miRNA-21, or other miRNAs, either with oncogenic or tumor suppressor roles.

Thus, the aims of the study were to: (a) compare the multipotency of CD44^+^ and CD44^−^ cells isolated from the same primary OSCC cultures by estimating their osteogenic and adipogenic differentiation capacity, (b) assess the effect of osteo- and adipo-induction on stemness-related genes’ expression, (c) analyze the influence of differentiation on apoptosis, and (d) investigate miR-21, miR-133, and miR-491 expression levels in CSCs during differentiation.

## 2. Results

### 2.1. CSCs’ Isolation and Characterization

**Figure 1:**
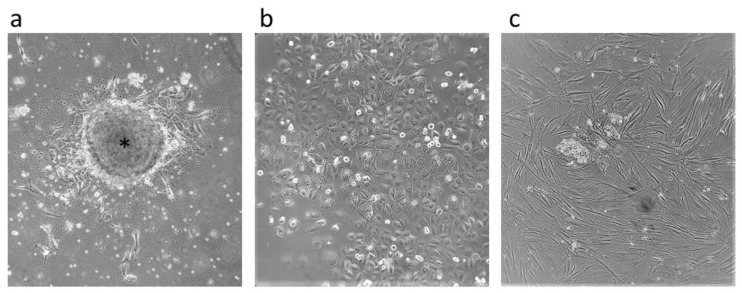
Cell cultures. Primary tumor cells arising from tumor tissue are indicated by an asterisk (**a**) and CD44-positive (**b**) and CD44-negative (**c**) cells after magnetic-activated cell sorting. All microphotographs are at 40× magnification.

**Figure 2:**
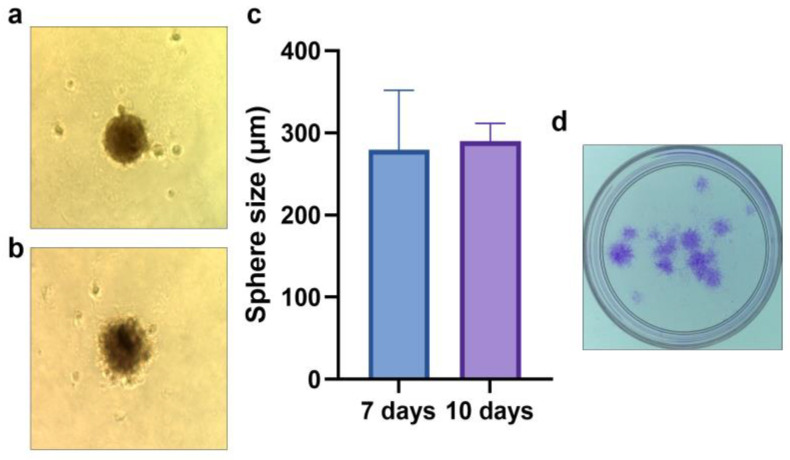
CSC properties of the isolated CD44^+^ cells–spheroid formation after 7 (**a**,**c**) and 10 days (**b**,**c**); colony forming assay after 7 days (**d**). Sphere microphotographs are at 40× magnification.

CD44^+^ positive cells were isolated from primary tumor cell cultures (Figure 1a) (i.e., separated from the remaining CD44^−^ cells) by a magnetic-activated cell sorting (MACS) system, and the further expanded (Figure 1b,c) MACS isolated cells were validated by flow-cytometry, resulting in over 92% of the population expressing CD44 (the data are not shown). Additionally, the cancer stemness properties of the isolated CD44^+^ cells were confirmed by the formation of tumor spheroids and colonies (Figure 2).

### 2.2. Osteogenic Differentiation

**Figure 3:**
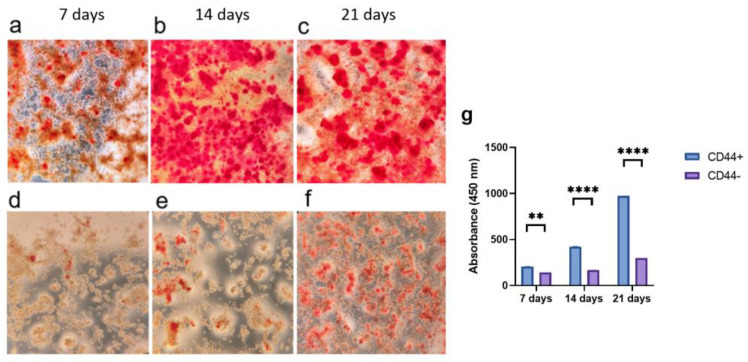
Osteogenic differentiation potential of CD44^+^ and CD44^−^ cells. Formation of mineralized nodules by CD44^+^ cells after 7 (**a**), 14 (**b**), and 21 days (**c**); and CD44^−^ cells after 7 (**d**), 14 (**e**), and 21 days (**f**). Quantification of Alizarin Red S staining (**g**). All microphotographs are at 40× magnification. CD44^+^—CD44-positive cells, CD44^−^—CD44-negative cells, ** *p* ≤ 0.01, and **** *p* ≤ 0.0001.

CD44^+^ and CD44^−^ cells, grown in an osteogenic differentiation medium for 7, 14, and 21 days, were stained with Alizarin Red S (Figure 3a–f), and the color was quantified (Figure 3g). The formation of mineralized nodules was significantly higher in the CD44^+^ compared to the CD44^−^ cell cultures after 7 (*p* = 0.0009), 14 (*p* = 0.00001), and 21 days (*p* = 0.00001) of cultivation (Figure 3g), pointing to a marked osteogenic differentiation potential of the CD44^+^ cells.

**Figure 4:**
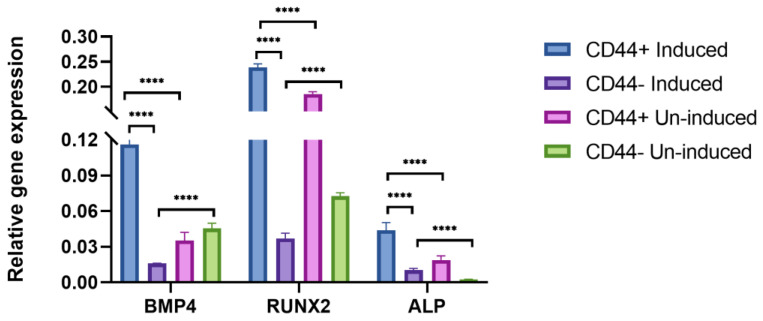
qPCR analysis of relative gene expression of osteogenic markers after 21 days of osteo-induction. BMP4, RUNX2, and ALP relative expression levels of CD44^+^ cells and CD44^−^ cells. Un-induced cells grown in a complete growth medium, and induced cells grown in the osteogenic medium. CD44^+^—CD44 positive cells, CD44^−^—CD44 negative cells, and **** *p* ≤ 0.0001.

In addition, to confirm the osteogenic differentiation after 21 days, *BMP4*, *RUNX2*, and *ALP* expression levels were assessed by qPCR (Figure 4). There were no major variations in the expression of the different osteogenic markers between the patients, as suggested by the relatively small standard deviations. The population of the CD44^+^ cells cultivated in the osteogenic medium showed a significantly higher expression of *BMP4* (a 3.3-fold increase, *p* ≤ 0.0001), *RUNX2* (a 1.3-fold increase, *p* ≤ 0.0001), and *ALP* (a 2.3-fold increase, *p* ≤ 0.0001) compared to the un-induced cells. Following the same conditions, the osteogenic induction was done in the CD44^−^ cell population. Compared to CD44^−^ cancer cells, the CD44^+^ cancer cell population has a substantially higher osteogenic differentiation potential, as judged from the relative expression levels of all the analyzed osteogenic markers (*p* = 0.0001).

**Figure 5:**
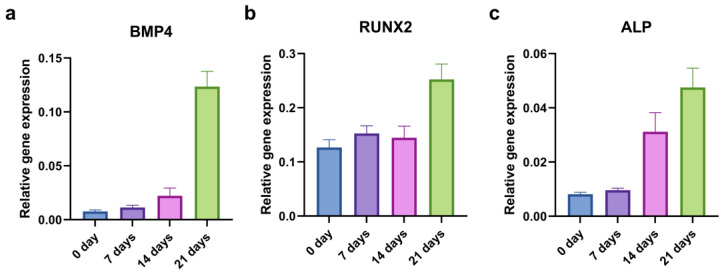
qPCR analysis of relative gene expression of osteogenic markers during 21 days of osteo-induction. *BMP4* (**a**), *RUNX2* (**b**), and *ALP* (**c**) relative expression levels in CD44^+^ cells.

To evaluate the kinetics of the osteogenic induction, we examined the relative gene expression of the *BMP4*, *RUNX2*, and *ALP* at the beginning (0 days) and after 7, 14, and 21 days of osteo-differentiation. Only the CD44^+^ cells were used in this experiment, and the levels of osteogenesis-related genes generally increased throughout time (Figure 5).

### 2.3. Adipogenic Differentiation of CD44^+^/CD44^−^ Cells

**Figure 6:**
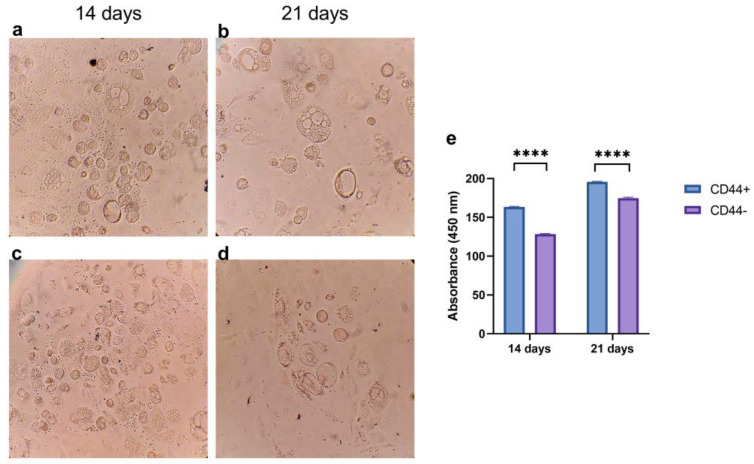
Adipogenic differentiation potential of CD44^+^ and CD44^−^ cells. Formation of neutral triglycerides and lipids by CD44^+^ cells after 14 (**a**) and 21 days (**b**); and CD44^−^ cells after 14 (**c**) and 21 days (**d**). Quantification of Oil Red O staining (**e**). All microphotographs are at 200× magnification. CD44^+^—CD44-positive cells, CD44^−^—CD44-negative cells, and **** *p* ≤ 0.0001.

CD44^+^ and CD44^−^ cells cultivated for 14 and 21 days in an adipogenic differentiation medium were stained using Oil Red O (Figure 6a–d), and the color was quantified (Figure 6e). The accumulation of neutral triglycerides and lipids was significantly higher in the CD44^+^ compared to the CD44^−^ cell cultures after 14 (*p* ≤ 0.0001) and 21 days (*p* ≤ 0.0001) of cultivation (Figure 6e). This demonstrated that the CD44^+^ cells were capable of adipogenic differentiation.

**Figure 7:**
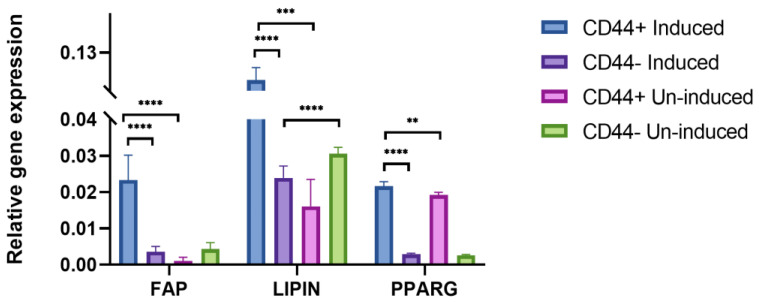
qPCR analysis of relative gene expression of adipogenic markers (*FAP*, *LIPIN*, and *PPARG*) after 21 days of adipo-induction. Un-induced cells grown in a complete growth medium, and induced cells grown in the osteogenic medium. CD44^+^—CD44-positive cells, CD44^−^—CD44-negative cells, ** *p* ≤ 0.01, *** *p* ≤ 0.001, and **** *p* ≤ 0.0001.

In addition, to confirm the adipogenic differentiation after 21 days, *FAP*, *LIPIN*, and *PPARG* expression levels were assessed by qPCR (Figure 7). Generally, there were no major variations in the expression of the different adipogenic markers between the patients, as suggested by the relatively small standard deviations. Populations of the CD44^+^ cells cultivated in the adipogenic medium showed a significantly higher expression of *FAP* (a 23.2-fold increase, *p* = 0.0001), *LIPIN* (a 7.6-fold increase, *p* = 0.002), and *PPARG* (a 1.2-fold increase, *p* = 0.002) compared to the un-induced cells. Following the same conditions, the adipogenic induction was conducted in the CD44^−^ cell population. Comparing the relative expression levels of all the adipogenic markers, a statistically significant difference was noted between the CD44^+^ and CD44^−^ cells (*p* = 0.0001), confirming their greater adipogenic differentiation potential based on the expression of specific markers after the adipogenic induction.

**Figure 8:**
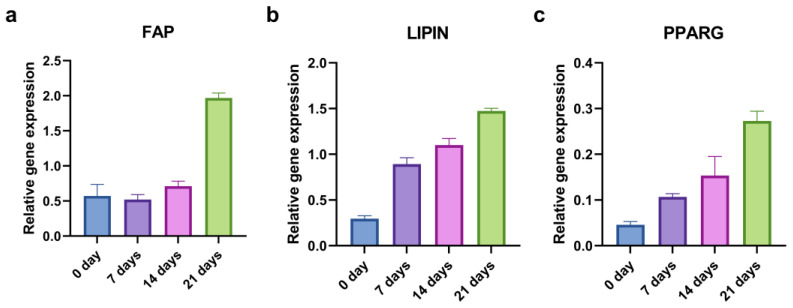
qPCR analysis of relative gene expression of adipogenic markers during 21 days of adipo-induction. *FAP* (**a**), *LIPIN* (**b**), and *PPARG* (**c**) relative expression levels in CD44^+^ cells.

To evaluate the kinetics of the adipogenic induction, we examined the relative gene expression of the *FAP*, *LIPIN*, and *PPARG* at the beginning (0 days) and after 7, 14, and 21 days of the adipo-differentiation. As for the osteogenic differentiation, only the CD44^+^ cells were used. The levels of adipogenesis-related genes generally increased throughout time (Figure 8).

### 2.4. Stemness Markers of CD44^+^ and CD44^−^ Cell Subpopulations

**Figure 9:**
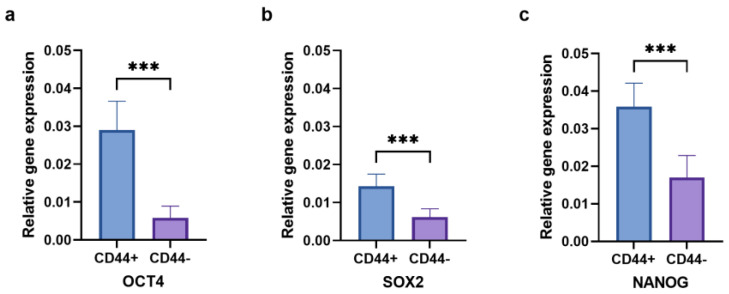
The expression of *OCT4* (**a**), *SOX2* (**b**), and *NANOG* (**c**) in CD44^+^ and CD44^−^ cells. CD44^+^—CD44-positive cells, CD44^−^—CD44-negative cells, and *** *p* ≤ 0.001.

The relative gene expression of the stem cell markers *OCT4* (Figure 9a), *SOX2* (Figure 9b), and *NANOG* (Figure 9c) was evaluated to confirm the cancer stem cell features of the CD44^+^ cells. The gene expression of all three markers was significantly higher in the CD44^+^ cells compared to the CD44^−^ cells (*p* ≤ 0.001).

### 2.5. Stemness Markers and Differentiation

**Figure 10:**
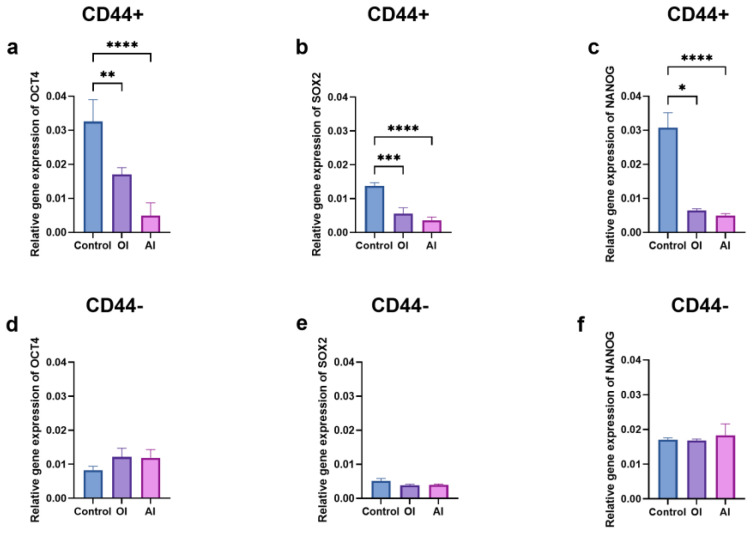
Relative gene expression levels of stemness markers in CD44^+^ and CD44^−^ cells after 21 days of osteo-induction (OI) and adipo-induction (AI). The expression of *OCT4* (**a**), *SOX2* (**b**), and *NANOG* (**c**) stem cell markers in CD44^+^; *OCT4* (**d**), *SOX2* (**e**), and *NANOG* (**f**) stem cell markers in CD44^−^ cells. CD44^+^—CD44-positive cells, CD44^−^—CD44-negative cells, * *p* ≤ 0.05, ** *p* ≤ 0.01, *** *p* ≤ 0.001, and **** *p* ≤ 0.0001.

To assess whether the cell differentiation led to alterations in the stem cell markers’ expression levels (*OCT4*, *SOX2*, and *NANOG*), the RNA was isolated from the cells grown in the osteogenic, adipogenic, or complete growth mediums for 21 days. The expression levels of the stem cell markers in the CD44^+^ cells were significantly lower (*p* ≤ 0.05) after the osteogenic (*OCT4*- 1.8, *SOX2*- 2.4, *NANOG*- 4.7–fold decrease) and adipogenic differentiations (*OCT4*- 6.5, *SOX2* -4.3, *NANOG* -6.1-fold decrease) compared to the un-induced, control cells (Figure 10a–c). The expression levels of the stem cell markers in the CD44^−^ subpopulation remained unchanged compared to the control (Figure 10d–f).

**Figure 11:**
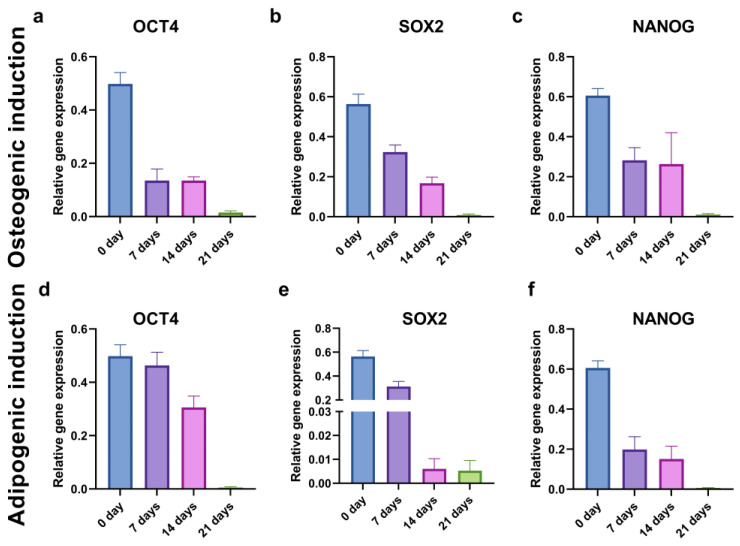
Relative gene expression levels of stemness markers *OCT4* (**a**,**d**), *SOX2* (**b**,**e**), and *NANOG* (**c**,**f**) in CD44^+^ cells during 21 days of osteo-induction (**a**–**c**) and adipo-induction (**d**–**f**).

To evaluate the kinetics of cancer stemness changes during the osteogenic and adipogenic inductions, we examined the relative gene expression of the *OCT4*, *SOX2*, and *NANOG* markers in the CD44^+^ cells at the beginning (0 days), and after 7, 14, and 21 days of differentiation. The levels of all the stemness-related genes decreased throughout time at a different pace (Figure 11).

### 2.6. Apoptosis after Differentiation

**Figure 12:**
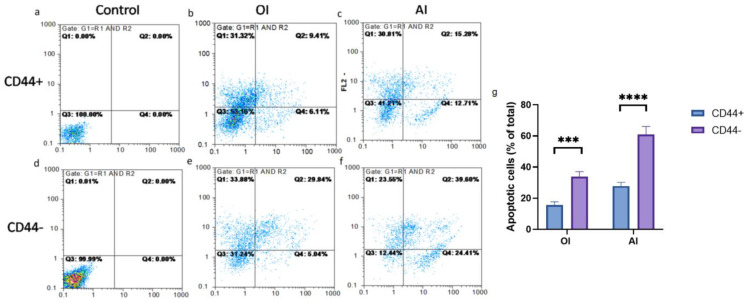
The effects of induction media on CD44^+^ and CD44^−^ cell viability. Annexin V apoptotic assay of un-induced CD44^+^ (**a**) and CD44^−^ cells (**d**); osteo-differentiated CD44^+^ (**b**) and CD44^−^ cells (**e**); adipo-differentiated CD44^+^ (**c**) and CD44^−^ cells (**f**); apoptotic cells (% of total cell number) upon osteo-induction (OI) and adipo-induction (AI) (**g**). Control cells cultured in a complete growth medium, CD44^+^—CD44-positive cells, CD44^−^—CD44-negative cells, *** *p* ≤ 0.001, and **** *p* ≤ 0.0001.

Upon the osteogenic and adipogenic differentiation inductions, considerable cell death by apoptosis was noted, but it was less pronounced in the CD44^+^ than the CD44^−^ cells (Figure 12). The percentage of apoptotic (early+late apoptosis) CD44^−^ cells was 34.88 after the OI and 64.01 after the AI (Figure 12g) and was almost 2 times higher compared to the CD44^+^ cells (15.52 after the OI and 27.99 after the AI) (Figure 12g).

### 2.7. Expression of miRNA-21, miRNA-133, and miRNA-491

**Figure 13:**
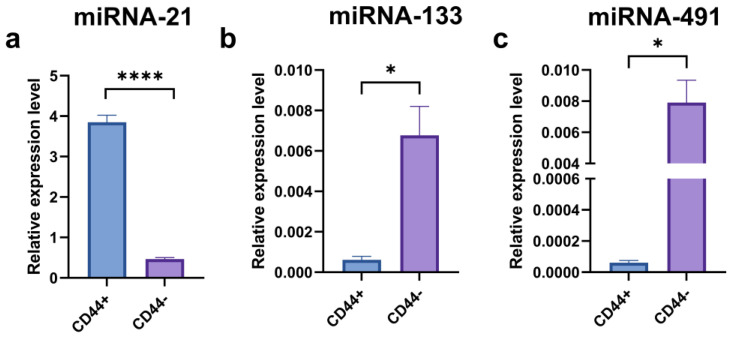
qPCR analysis of relative miRNA-21 (**a**), miRNA-133 (**b**), and miRNA-491 (**c**) expressions in CD44-positive versus CD44-negative cells; * *p* ≤ 0.05, **** *p* ≤ 0.0001.

A significantly higher expression level of miRNA–21 was observed in the CD44^+^ compared to the CD44^−^ cell cultures (Figure 13a) (*p* = 0.0008). The CD44^+^ cell differentiation influenced the expression level of miRNA-21. On the contrary, the miRNA-133 and miRNA-491 expression levels were significantly (*p* < 0.05) higher in the CD44^−^ compared to the CD44^+^ cell culture.

**Figure 14:**
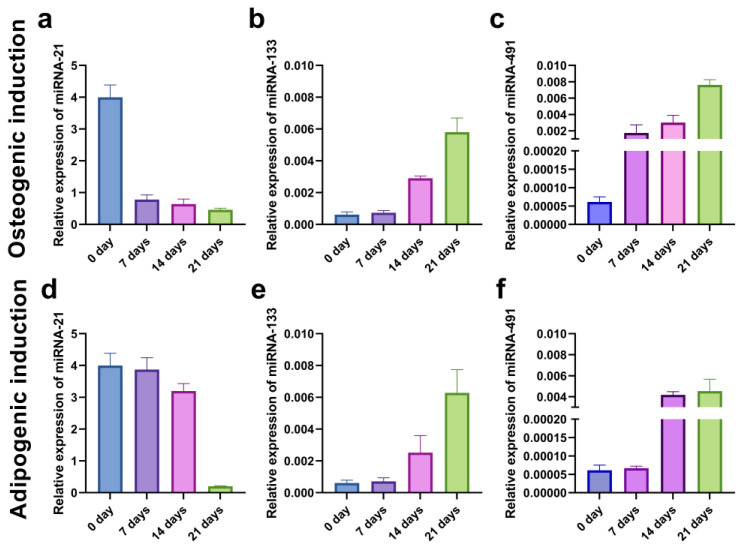
qPCR analysis of relative miRNA-21 (**a**,**d**), miRNA-133 (**b**,**e**), and miRNA-491 (**c**,**f**) expressions in CD44^+^ cells from the SCC-25 during 21 days of osteo-induction (**a**–**c**) and adipo-induction (**d**–**f**).

To evaluate the kinetics of the miRNA expression pattern during the osteogenic and adipogenic inductions, we examined the relative expression of miRNA-21, miRNA-133, and miRNA-491 in the SCC-25 cell line at the beginning (0 days) and after 7, 14, and 21 days of differentiation. The level of oncogenic miRNA-21 decreased throughout time, while the expression of miRNA-133 and miRNA-491 increased, suggesting their role as tumor-suppressors (Figure 14).

## 3. Discussion

CSCs represent a subpopulation of pluripotent cells in cancer that possess a high proliferative and self-renewal capacity. In vitro, CSCs grow faster than normal cells and have a high colony formation ability. They can be separated by using specific biomarkers, mostly located on the cell surface, such as CD44, CD133, EpCAM, etc. [18]. In this study, the characteristic marker, CD44, and magnetic-activated cell sorting were used to isolate the CSCs (CD44^+^ cells) from primary OSCC cell cultures.

The main objective of our study was to investigate the capacity of CSCs to undergo differentiation into specific lineages and, to the best of our knowledge, this is the first report dealing in parallel with the osteogenic and adipogenic potential of CSCs originating from OSCC.

In the present study, it was clearly shown that the CD44^+^ subpopulation exhibited a much higher potential for osteogenic and adipogenic differentiation compared to CD44^−^, as judged from the specific staining and expression of the respective markers. This finding is consistent with the stemness characteristics of CD44^+^ cells. Namely, the expression of embryonic stem cell markers, *OCT4*, *SOX2*, and *NANOG*, which play a crucial role in stemness maintenance and cell migratory capacity, was significantly higher in the CD44^+^ than in the CD44^−^ cell populations. Concomitantly, with the increase of specific, either osteogenic or adipogenic markers, there was a sharp decrease in the embryonic stemness markers, a logical phenomenon accompanying the process of cell differentiation. The process of differentiation was monitored throughout time in the CD44^+^ cells, and a relatively gradual decrease of embryonic markers followed the similarly even increase of lineage-specific markers. Patil et al., in a recent study, demonstrated the capacity of OSCC CD44^+^ cells to undergo adipogenic differentiation and showed increased levels of adipogenic markers, concomitantly with the decrease of stemness markers (*SOX2*, *NANOG* and Kruppel-like factor 4—*KLF4*), which is fully in line with our findings [19]. Our findings are also in line with the findings reported by Zhau et al., who observed a decreased expression of stemness markers upon adipogenic induction in the human prostate cancer cell line (PC–3 cells) [6]. Milosevic et al. also noted a decrease of *OCT4*, *SOX2*, and *NANOG* expressions following the osteo- and chondro-induction of basal cell carcinoma CSCs [5].

The concept of CSC differentiation as an antineoplastic therapy is becoming increasingly appealing. Indeed, the process of differentiation induction results ultimately in the terminal differentiation of CSCs, which in turn irreversibly abolishes their tumorigenic potential and proliferative ability, causing them to become sensitive to conventional anticancer treatments [20,21].

Interestingly, the osteogenic and adipogenic inductions affected cell viability, implying that mature cells have a limited lifespan and eventually perish through programmed cell death mechanisms, mostly through apoptosis [22]. In the present study, adipogenic induction led to a 64% and 29% apoptosis rate in CD44^−^ and CD44^+^ cell subpopulations, respectively, while osteogenic differentiation had a lesser impact on apoptosis. Zhau et al. noted that adipogenic induction led to the apoptosis of 77% of the PC–3 cell population [6].

New evidence, especially regarding miRNAs and CSCs, has completely changed our understanding of carcinogenesis. Small noncoding molecules (miRNAs) are considered to control the expression of more than 60% of human genes. The aberrant expression of miRNAs has been linked to the development of human malignancies and the regulation of stemness properties of CSCs [23,24]. The current findings indicate that changes in the expression of miRNAs are related to tumor functions [25].

In our study, we examined the levels of three micro RNAs: one oncogenic (miR–21) and two tumor suppressors (miRNA-133 and miRNA-491) in CD44^+^ and CD44^−^ cells. It appeared that CD44^+^ cells had a significantly higher miRNA-21 expression and significantly lower miRNA-133 and miRNA-491 than the CD44^−^ cells. Then, we examined the levels of all three micro RNAs during the process of differentiation, both osteo- and adipogenic in the CD44^+^ cells. We established that changes in the levels of micro RNAs followed the expected kinetics. Namely, in parallel with the differentiation process and accompanied by the loss of stemness properties, a significant decrease of miRNA-21 was registered. This is the first report indicating that miRNA-21 expression levels decrease in the OSCC CSCs’ subpopulation during osteo/adipo-differentiation. Our findings support the concept of miRNA-21 involvement in the maintenance of oral cancer CSCs’ stemness. Namely, this oncomiRNA has consistently been linked to colon and pancreatic CSCs’ regulation [26,27]. In anaplastic thyroid carcinoma therapy, the knockdown of miRNA-21 has significantly impacted the expression pattern of genes involved in the control of stemness, tumor growth, differentiation, and apoptosis [28].

On the contrary, levels of both the micro RNAs with tumor suppressor activity (miRNA-133 and miRNA-491) increased over time and the course of differentiation. Our findings are in agreement with those of Huan et al., who established that miRNA-491 was a modulator of OSCC behavior and that lower levels of this micro RNA were related to poorer survival, confirming its tumor suppressor role [29]. Similarly, He et al. showed that miRNA-133 was able to restrain OSCC proliferation and invasion, again corroborating its tumor suppressor role, which is fully in agreement with our findings [30].

Altogether, these findings suggest that differentiation leads to the loss of malignant phenotypes and the appearance of terminally differentiated cells with seemingly “normal” adult cell phenotypes.

However, there are several limitations to our study. All the experiments have been conducted in vitro; therefore, it was not possible to assess how the microenvironment would affect the CSCs’ differentiation and the micro RNAs’ expression. In the near future, it will be necessary to examine in more detail the signaling pathways involved in OSCC CSC stemness and their relationship with different micro RNAs’ expression during differentiation.

In conclusion, we have successfully isolated CD44^+^ cells from primary OSCC cell cultures and successfully induced osteogenic and adipogenic differentiation. The process of differentiation has affected the CD44^+^ stemness properties, their viability, and the levels of both oncogenic and tumor suppressor micro RNAs, implying that CSC differentiation might be used as a novel therapeutic modality.

## 4. Materials and Methods

### 4.1. Cell Cultures

In the present study, primary cell cultures were generated from tumor tissue samples of five patients diagnosed with OSCC (3 males and 2 females, aged 59.2 ± 8.04 years, localization—tongue, the floor of the mouth, and gingiva; three patients had T2N0M0, and two had T4aN0M0 status; two patients had bone infiltration, and all of them had an HG2 NG2 tumor grade) obtained immediately after surgery from the Clinic of Maxillofacial Surgery of the School of Dental Medicine, University of Belgrade. All patients were informed of the study and signed a written informed consent form. All samples were examined by a pathologist, and the diagnosis of oral squamous cell carcinoma was confirmed. None of the patients recruited in this study received any preoperative chemotherapy or radiotherapy. The study was approved by the institutional Ethical Committee (No 36/6) of the University of Belgrade, Republic of Serbia, in accordance with the Declaration of Helsinki. Tissue samples were cut with blades into small pieces and washed three times with phosphate-buffered saline (PBS), then seeded into T25 cell culture flasks in a complete growth medium (Dulbecco’s Modified Eagle Medium (DMEM), supplemented with 10% of Fetal Bovine Serum (FBS), 100 U/mL of a penicillin–streptomycin solution, and 400 ng/mL hydrocortisone, all chemicals purchased from Invitrogen, Thermo Fisher Scientific, Waltham, MA, USA) under standard conditions in a humidified atmosphere with 5% CO_2_ at 37 °C. The complete growth medium was changed every 2–3 days, and after the cells reached 80% of confluence, the heterogenic cell population was magnetically sorted. In addition, the SCC–25 (ATCC^®^, CRL– 1628™) cell line was also used for miRNA kinetics experiments. The culturing conditions of the SCC–25 cell line were the same as for primary cultures.

### 4.2. Magnetic-Activated Cell Sorting

CD44^+^ cell separation from primary cultures and the SCC–25 cell line was performed using a magnetic-activated cell sorting (MACS) system (Miltenyi Biotec, San Francisco, CA, USA) according to the manufacturer’s protocol. Total populations of adherent cells (2 × 10^6^) were enzymatically detached and counted. The cells were incubated with 100 μL of CD44 magnetic microbeads (Miltenyi Biotech) at 4 °C for 30 min. Upon incubation, the cell suspension was passed through the MACS MS column and placed in the magnetic field of a MACS separator. CD44-positive (CD44^+^) cells were retained in the column, and the unlabeled cells were eluted as a suspension known to consist of CD44-negative (CD44^−^) cells and seeded onto a new T25 flask [31]. When the column was removed from the magnetic field, the magnetically retained cells (CD44^+^) were also seeded into another flask for further experiments.

### 4.3. Spheroid Formation Assay

As previously described [9], CD44^+^ cells were seeded at a density of 10^3^/mL on 24-well culture plates that had been coated with 1ml poly-HEMA (poly 2−hydroxyethyl methacrylate, Sigma−Aldrich, Taufkirchen, Germany) to inhibit cell attachment. Cells were cultured in DMEM supplemented with B-27, N2, an epidermal growth factor, and antibiotics following germination (Sigma−Aldrich). Following 7 and 10 days of incubation, the size of spheroids was determined using ImageJ software 1.48 version (NIH, Bethesda, MD, USA) (Java 1.8.9_66).

### 4.4. Colony-Forming Assay

To determine the clonogenic potential of CD44^+^ cells, a colony-forming assay was performed, as previously described [32]. Briefly, CD44^+^ cells were seeded in a 32mm Petri dish at a density of 1000 cells/plate. After 7 days, the colonies were stained with 0.1% of a Coomassie Blue solution (Sigma−Aldrich).

### 4.5. Differentiation Capacity of CD44^+^ and CD44^−^ Cells

CD44^+^ and CD44^−^ cells were seeded onto 24-well culture plates (1 × 10^4^ per well) and cultured in the complete growth medium. After reaching 80% of confluence, the cells were cultivated in an optimized medium for osteogenic and adipogenic differentiation, respectively (StemMACS™, Miltenyi Biotec) for 7, 14, and 21 days. Cells were incubated under standard conditions (5% CO_2_ at 37 °C), and the medium was replaced every 3rd day. Cells were washed twice with PBS, then fixed with a 4% paraformaldehyde solution for 30 min at room temperature. An Alizarin Red S 2% solution (Centrohem, Belgrade, Serbia) for osteogenic differentiation and a 0.5% Oil Red O solution (Sigma−Aldrich) for adipogenic differentiation were poured over the cells, and the samples were incubated for 30 min and washed with distilled water. To quantify the calcium deposits and lipid droplets in the matrix, 10% acetyl pyridinium chloride was added for de-staining. The absorbance of the solution was measured at 450 nm OD using a microplate reader. The quantification was normalized against the stained cells grown in the complete growth medium [33].

### 4.6. Apoptosis Assay

CD44^+^ and CD44^−^ cells were seeded into 24-well plates (1 × 10^5^ per well) and cultured in respective differentiation media (1 mL of medium per well). After 14 days of induction, Annexin staining for detecting apoptosis was performed with an Annexin V–FITC Apoptosis Detection Kit (Invitrogen, Thermo Fisher Scientific) according to the manufacturer’s instructions. Annexin V–FITC staining was analyzed by flow cytometry, and the results were presented in a two-dimensional dot plot of propidium iodide (PI) versus Annexin V–FITC. PI was used to detect necrotic or late apoptotic cells. The plots were divided into four regions corresponding to: (a) viable cells, negative for both probes (PI/FITC −/−; Q3); (b) apoptotic cells, PI-negative and Annexin-positive (PI/FITC −/+; Q1); (c) late apoptotic cells, PI- and Annexin-positive (PI/FITC +/+; Q2); (d) necrotic cells, PI-positive and Annexin-negative (PI/FITC +/−; Q4). The cells cultured in the complete growth medium under the same conditions were used as controls.

### 4.7. RNA Isolation and Reverse Transcription

Total RNA was extracted from the 21-day-old osteo- and adipo-induced cells using a TRIzol Reagent (Invitrogen, Thermo Fisher Scientific), according to the manufacturer’s recommendations. The RNA concentration was measured using a microvolume spectrophotometer (BioSpec–nano Microvolume UV–Vis Spectrophotometer; Shimadzu Scientific Instruments, Columbia, MD, USA). An oligo d(T) primer and RevertAid First Strand cDNA Synthesis Kit (Thermo Fisher Scientific, Waltham, MA, USA) were used to synthesize cDNA from 2 µg of total RNA [34]. For assessing the miRNA–21 expression level in OSCC tissues, RNA was isolated from CD44^+^ and CD44^−^ cell cultures.

### 4.8. Gene Expression Analysis of Differentiation and Stemness Markers

Real-time quantitative polymerase chain reaction (qPCR) was performed using the first strand cDNA, 0.2 μM forward and reverse primers, and a SensiFAST SYBR Hi–ROX Kit (Bioline, London, UK). The expression of the following markers was analyzed: *ALP*, *RUNX2*, *OCN*, and *BMP2* (osteogenic) and *PPARG*, *FAP*, and *LIPIN* (adipogenic). In addition, the expression of embryonic stem cell markers (*OCT4*, *SOX2*, and *NANOG*) was also examined, after the CSCs’ separation and differentiation. The housekeeping gene, glyceraldehyde-3-phosphate dehydrogenase—*GAPDH*, was used as a reference. Relative gene expression values were calculated using the 2−ΔCt method [35]. The sequences of all primers used in this study are given in Table 1.

### 4.9. TaqMan microRNA Assay

Reverse transcription was accomplished using 15 ul reactions that consisted of 10× a Reverse Transcription Buffer, an RNase inhibitor, 100 mM deoxyribonucleotide triphosphate (dNTP), and a Multi Scribe Reverse Transcriptase, and containing 3 μL of a 5× concentrate miRNA-21-, miRNA-133-, and miRNA-491-specific primers. Thermal cycler settings were 16 °C for 30 min, 42 °C for 30 min, and 85 °C for 5 minutes, followed by cooling to 4 °C. Quantitative polymerase chain reaction (qPCR) was accomplished in 20 μL reactions using a TaqMan 20× concentrate of miRNA-21, miRNA-133, and miRNA-491 assays (all from Applied Biosystems, Thermo Fisher Scientific, Waltham, MA, USA), Universal PCR Master Mix, and the product from the reverse transcription reaction. Thermal cycler settings were 50 °C for two minutes, 95 °C for 10 minutes, then 40 cycles of 95 °C for 15 s and 60 °C for 60 s.

The fold change was calculated based on the threshold cycle (Ct) value using the formula: Relative Quantity (RQ) = 2^−ΔΔCT^, using RNU44 as the internal control.

### 4.10. Statistical Analysis

The data analysis was performed using the statistical software GraphPad Prism version 9.0 (GraphPad Software, Inc., La Jolla, CA, USA). The normality of the distribution was confirmed by a Kolmogorov–Smirnov test. To identify statistical differences between groups, a one-way ANOVA test was applied, followed by Dunnett’s multiple comparison test. The difference was considered statistically significant when *p* ≤ 0.05. All experiments were conducted in triplicate and repeated at least two times.

## Figures and Tables

**Table 1:** Primers with corresponding sequences used in the study.

*OCT4*	Rv	5′ TGC TCC AGC TTC TCC TTC TC 3′
	Fw	5′ GTG GAG AGC AAC TCC GAT G 3′
*SOX9*	Rv	5′ CTC TTT TGC ACC CCT CCC ATT 3′
	Fw	5′ GAC TTC ACA TGT CCC AGC ACT A 3′
*NANOG*	Rv	5′ TTT TTG CGA CAG TCT TCT CTG C 3′
	Fw	5′ ATT CAG GAC AGC CCT GAT TCT TC 3′
*RUNX2*	Rv	5′ GTC TCG GTG GCT GGT AGT GA 3′
	Fw	5′ ACA AAC AAC CAC AGA ACC ACA AGT 3′
*ALP*	Rv	5′ ATG GCA GTG AAG GGC TTC TT 3′
	Fw	5′ CCA CGT CTT CAC ATT TGG TG 3′
*BMP4*	Rv	5′ GGG TGA GTG GAT GG AAC 3′
	Fw	5′ CTC GAT GAG TAT GAT AAG GTG GTA 3′
*FAP*	Rv	5′ TTA TGC TCT CTC ATA AAC TCT CGT GGA ATG GAC 3′
	Fw	5′ ATG TGT GAT GCT TT GTA GGT ACC TG 3′
*LIPIN*	Rv	5′ TGG ACT CTT TCA TCT TGT GTG GA 3′
	Fw	5′ CCC GAC CTT CAA CAC CTA AAA GT 3′
*PPARG*	Rv	5′ TGG ATC TGT TCT TGT GAA TG 3′
	Fw	5′ CGT GGA TCT CTC GGT AAT 3′
*GAPDH*	Rv	5′ CCC TGT TGC TGT AGC CAA ATT CGT 3′
	Fw	5′ TCA TGA CCA CAG TCC ATG CCA TCA 3′

## Data Availability

Not applicable.
